# Hypothalamic L-Histidine Decarboxylase Is Up-Regulated During Chronic REM Sleep Deprivation of Rats

**DOI:** 10.1371/journal.pone.0152252

**Published:** 2016-12-20

**Authors:** Gloria E. Hoffman, Michael Koban

**Affiliations:** Department of Biology, Morgan State University, Baltimore, Maryland, United States of America; Kent State University, UNITED STATES

## Abstract

A competition of neurobehavioral drives of sleep and wakefulness occurs during sleep deprivation. When enforced chronically, subjects must remain awake. This study examines histaminergic neurons of the tuberomammillary nucleus of the posterior hypothalamus in response to enforced wakefulness in rats. We tested the hypothesis that the rate-limiting enzyme for histamine biosynthesis, L-histidine decarboxylase (HDC), would be up-regulated during chronic rapid eye movement sleep deprivation (REM-SD) because histamine plays a major role in maintaining wakefulness. Archived brain tissues of male Sprague Dawley rats from a previous study were used. Rats had been subjected to REM-SD by the flowerpot paradigm for 5, 10, or 15 days. For immunocytochemistry, rats were transcardially perfused with acrolein-paraformaldehyde for immunodetection of L-HDC; separate controls used carbodiimide-paraformaldehyde for immunodetection of histamine. Immunolocalization of histamine within the tuberomammillary nucleus was validated using carbodiimide. Because HDC antiserum has cross-reactivity with other decarboxylases at high antibody concentrations, titrations localized L-HDC to only tuberomammillary nucleus at a dilution of ≥ 1:300,000. REM-SD increased immunoreactive HDC by *day* 5 and it remained elevated in both dorsal and ventral aspects of the tuberomammillary complex. Our results suggest that up-regulation of L-HDC within the tuberomammillary complex during chronic REM-SD may be responsible for maintaining wakefulness.

## Introduction

In a general sense, higher animals are in one of two behavioral states: awake or asleep, with rapid transitions between them [1]. Sleep or a state comparable to mammalian sleep is highly conserved, being found across a remarkably wide range of phylogenies [2]. Despite there being a biological need for sleep or a sleep-like state, its evolutionary underpinnings and functions for homeostasis remain elusive [3], although restoration of brain energy balance by sleep has been championed [4, 5].

That sleep is physiologically important is best exemplified by depriving an animal of sleep and observing the consequences during continued wakefulness. Several deprivation methods have been employed using rats. Gentle handling or placement of novel objects in the cage are suitable for several hours of deprivation [6]. But when deprivation is chronically applied for days or weeks, other techniques are used. For example, the inverted flowerpot involves a rat residing on a small circular platform surrounded by water [7]. When the animal enters REM sleep, muscle atonia causes facial or bodily contact with the water and awakens the animal, thereby continuously reinforcing the paradigm. This method specifically abolishes REM sleep and reduces non-REM sleep by almost 40% over 96 hours [8]. The disk-over-water (DOW) method uses an experimental rat instrumented for EEG monitoring. When it enters a specific sleep stage (e.g., REM sleep), a motor-driven disk rotates and the rat must awaken to keep from being pushed into the water [9, 10]. Others have used forced locomotion of rats in slowly rotating drums [11].

Chronic loss of sleep causes the rat to manifest a number of pathologies or syndromes [10, 12]. For instance, profiles of circulating hormones change [13–16], energy expenditure and metabolism increase [17–19], hypothalamic neuropeptides governing appetite and the stress response are altered [20, 21], and rats become hyperphagic but do not gain body weight [15, 18, 21–23]. In a series of studies with the DOW method, Rechtschaffen and colleagues [9, 10] determined that it does not matter whether it is REM sleep [24] or total sleep [25] that is being deprived: the same pathologies develop in both, albeit with different time courses. Moreover, it is not possible to adapt to sleep deprivation (at least in rats) because progressive morbidity always culminates in death after about 20 days [10, 24, 25].

Sleep and wakefulness are active processes involving separate but interrelated systems that are normally in rhythmic balance yet enormously complex. The ascending arousal system from the brainstem through the thalamus, hypothalamus, basal forebrain, and cerebral cortex involve adrenergic, cholinergic, dopaminergic, histaminergic, GABAergic, glutaminergic, and other neurotransmitters, as well as peptidergic neurons that release orexins/hypocretins, melanin concentrating hormone, and galanin. Moreover, Blanco-Centurion et al. [26] provide evidence that norepinephrine and histamine are important in REM sleep control based on saporin-neurotoxin lesions placed in locus coeruleus and tuberomammillary complex, respectively. Thus, it is logical that these two systems would be required for the maintenance of wakefulness during sleep disruption.

The mode of transmitter regulation for the monoamines relies, in part, upon the changes in expression of the rate-limiting enzymes of biosynthesis and is well established for noradrenergic, dopaminergic, and serotinergic control. For example, with enforced wakefulness, it is adaptive that the rate-limiting enzyme for norepinephrine synthesis, tyrosine hydroxylase, is up-regulated in the locus coeruleus [27, 28]. The posterior hypothalamus, and specifically the tuberomammillary nucleus (TMN), is the location of histamine neurons [29, 30]. It is now recognized that histamine has a major function involving wakefulness [31]: it is higher in CSF during the dark phase when animals are awake, after application of drugs that promote wakefulness, and after 6 h of sleep deprivation [32]. The most obvious and well-known example of histamine’s role in wakefulness and sleep is the sedative effect of first-generation antihistamine drugs (diphenhydramine, an H1 receptor antagonist [e.g., Benadryl]) [33, 34].

In the present study, we tested the hypothesis that L-histidine decarboxylase (HDC; EC 4.1.1.22), the enzyme responsible for histamine biosynthesis in TMN neurons of the posterior hypothalamus, would be up-regulated during chronic and unremitting REM sleep deprivation (REM-SD) of rats. Two lines of reasoning and observations were considered to support our hypothesis. First, previously we showed that REM sleep-deprived rats progressively increase rates of resting oxygen consumption concomitant with a robust increase in uncoupling protein 1 (UCP1) gene expression in brown adipose tissue (BAT) [19]. Regulation of UCP1-mediated thermogenesis by sympathetic nerve activity is well established [35], and with the findings by Yasuda and colleagues [36, 37] that administration of histamine into the paraventricular nucleus or preoptic area, or central or peripheral injection of L-histidine, the precursor to histamine synthesis, results in significant stimulation of sympathetic nerve activity to BAT and increases UCP1 mRNA, we rationalized that there would be an increase in HDC within TMN neurons. Secondly, because the REM-SD paradigm (the inverted flowerpot or platform-over-water or method) enforces increased wakefulness for many days (albeit with some fragmented slow-wave sleep) [8], this suggested that the wakefulness centers, and specifically histaminergic neurons, must be more active.

## Materials and Methods

All experimental procedures were approved by the Institutional Animal Care and Use Committee of Morgan State University (IACUC protocol RA-05-B-210), and they comply with the Guide for the Care and Use of Laboratory Animals of the National Institutes of Health.

The tissues used in this study were from a cohort of rats of an earlier study [20]. Briefly, at each REM-SD or control time point, rats were anesthetized with an overdose of sodium pentobarbital (100 mg/kg, ip), administered heparin (100 U) directly into the heart, and perfused transcardially with saline containing 2% sodium nitrite followed by 2.5% acrolein in buffered 4% paraformaldehyde [38]. The brains were removed and sunk in 30% sucrose solution, frozen, and sectioned at 25 μm on a freezing sliding microtome into 1-in-12 series. The sections were collected in cryoprotectant anti-freeze solution [39]. Sections were stored at –20°C until they were processed for immunocytochemical analysis. This procedure enables reliable maintenance of immunoreactivity for >12 years [39, 40].

### Chronic REM Sleep Deprivation (REM-SD) Paradigm

REM-SD of male Sprague-Dawley rats (4–5 months of age; Harlan Laboratories) was enforced with the inverted flowerpot or platform-over-water method [20]. Two Plexiglas tanks are each divided into 5 chambers (30 X 30 X 40 cm). Within each chamber, a rat resides on a 10-cm diameter platform with easy access to food (Harlan Laboratories, TekLad Rodent Diet 8604) and water bottle; it can engage in grooming and some exploratory behaviors. Photoperiod was 12:12 with lights-on at 0900 h. Warm water (~30°C) floods each chamber to about 1 cm below the platform and the water flow-through system continuously carries away waste and food debris. Rats were acclimated to the sleep deprivation tank for 1 hour per day for at least 2 weeks. Enforcement of the paradigm begins with slow-wave sleep becoming fragmented. As the rat enters REM sleep, muscle atonia causes facial or bodily contact with the water; it immediately awakens, and the cycle continues. Machado et al. [8] used this method for 96 h to validate by EEG that REM sleep is completely abolished and that slow wave sleep significantly decreases. In our earlier study [20], rats were REM sleep-deprived for 5, 10, 15, and 20 days; controls were rats in their home cages. Because remaining brain tissues were limited for the current study of HDC expression in the TMN, different rostral and caudal sections were required. Initially, we had not intended to keep and analyze sections at the most caudal extent of the hypothalamus where the ventral TMN (vTMN) is located. Thus, analysis of this area was possible only for some of the animals, where vTMN had *n* = 4 controls and *n* = 4 each for REM-SD *day* 5 and 10; there was insufficient tissue for examination of vTMN of *day* 15 or 20 REM sleep-deprived rats. Analysis of the dorsal TMN (dTMN) had *n* = 4 controls; for REM-SD *days* 5, 10, and 15, there were *n* = 6, 5, and 2 rats, respectively. For the 2 rats euthanized on *day* 15, brain tissue provided only qualitative immunocytochemistry (ICC) data for comparative visual purposes.

Some investigators include REM-SD tank controls where the platform size is sufficiently large (e.g., 15-cm) to allow rats to sleep [8]. In our previous study on effects of chronic REM-SD on energy metabolism [19], tank controls showed no differences in any of the parameters evaluated compared to rats kept in their home cages, and thus for this study, only home cage rats were used as controls. Moreover, analysis of the literature of rat flowerpot REM-SD studies show no significant effect of using tank controls.

### Immunocytochemistry for Histamine and L-Histidine Decarboxylase

Primary antibodies were rabbit anti-L-histidine decarboxylase (HDC) antiserum (PROGEN Biotechnik; catalog number 16045) and rabbit anti-histamine antiserum (ImmunoStar; catalog number 22939). In rodents, one population of neurons in the TMN contains the neurotransmitter histamine [29, 30] and expresses HDC, the enzyme responsible for its synthesis [41]; HDC mRNA is detected in just that one location [42, 43]. It was critical for this study to be certain that the antiserum and the methods were examining changes in just HDC with no cross-reactivity to other decarboxylases that have been reported [44–46]. We began by validating the localization of histamine. Since anti-histamine antibody will only recognize the antigen when tissue is fixed with *N-*(3-dimethyl-aminopropyl)-*N’*-ethyl carbodiimide hydrochloride (i.e., carbodiimide; EDAC; Sigma-Aldrich, catalog number E7750), followed by paraformaldehyde [47], the strategy was to first select carbodiimide-fixed brain sections and examine the cell patterns for staining of histamine. A new cohort of control rats (*n* = 7) was perfused and fixed with 4% carbodiimide followed by buffered 5% paraformaldehyde for this trial and compared with tissue from other rats (*n* = 4) fixed with 2.5% acrolein and 4% paraformaldehyde, the combination of which is optimal for protein ICC [38, 48]. In addition, several brains were fixed with only 4% paraformaldehyde as a negative control for histamine but were useful for enzyme localization. The purpose of this was to verify that anti-histamine antiserum shows no immunoreactivity if 4% paraformaldehyde is the only fixative, and to assess whether addition of acrolein to the fixative maintained histamine immunoreactivity as would be expected for HDC. The brains were removed, blocked to isolate the hypothalamus, sunk in 30% aqueous sucrose, cut on a freezing microtome at 25 μm, and stored in cryoprotectant antifreeze solution [39, 40]. Antisera were diluted in 0.05M potassium phosphate buffered saline (pH 7.4) with 0.4% Triton-X 100 (Sigma-Aldrich), and titrations were conducted to determine the best dilution [49]. For anti-histamine antiserum, titrations were done from 1:1000 to 1:300,000 in half-logarithmic steps. The optimal dilutions were 1:15,000–20,000 for saturating staining and 1:100,000 for graded staining of histamine. For anti-HDC antiserum, a range of antibody dilutions between 1:3000 and 1:700,000 in approximate half-logarithmic steps were used to assess whether HDC could be localized without cross-reactivity within non-histaminergic structures known to express L-amino acid decarboxylase. Secondary antibody was biotinylated goat anti-rabbit IgG (1:600, heavy- and light chain-specific; Vector Laboratories). Immune complexes were incubated with ABC reagents (Vector Laboratories, Vectastain kit, 4.5 μL/mL of solution A and B; catalog number PK-6100) and nickel sulfate-diaminobenzidine (NiDAB:25 mg/mLNiSO_4_ • 6H_2_O [Sigma Aldrich, catalog number N4882], 0.2 mg/mL of 3,3’-diaminobenzidine tetrahydrochloride hydrate [Sigma-Aldrich Fluka, catalog number 32750] made in 0.175M sodium acetate [Fisher Scientific], and 0.83 μL/mL of 3% H_2_O_2_ [any local pharmacy]) as the chromogen. Staining times were held constant at 15 minutes [49], after which sections were mounted onto subbed slides, dried overnight, dehydrated through an ascending series of alcohols, cleared in xylene and cover-slipped using Histo-Clear (National Diagnostics, catalog number HS-200).

### Data Presentation and Analyses

After establishing HDC optimal assay conditions, the slides were coded so that the individual conducting the analysis was blind to the condition of the animals. Images of histaminergic neurons from the population located immediately lateral to the dorsal third ventricle at its posterior extent (dorsal tuberomammillary nucleus, dTMN) or the more caudal and ventrolateral population (ventral tuberomammillary nucleus, vTMN) were captured at 100X with a cooled Retiga CCD digital camera using iVision software (BioVision Technologies). Sections containing the vTMN were selected and images were captured using a constant exposure time. The staining intensity of all the TMN immunoreactive neurons was measured and a region outside of the TMN where no histaminergic-immunoreactive cells or axons (based on saturated histamine staining) are located was used to assess “noise” or non-specific staining. This value was subtracted from the cell gray levels. Throughout, darkness refers to intensity of staining of immunoreactive HDC grey level minus background grey levels (when eliminating the primary or using of such low concentrations of primary antibody that no reaction product was observed). Statistical analysis (*P* ≤ 0.05 being significant) used the t-test (GraphPad Prism [GraphPad Software, version 5.04 for Windows]). Data are shown as mean ± SEM.

## Results

### Defining Localization of Histamine in the Tuberomammillary Nucleus (TMN)

**Fig 1:**
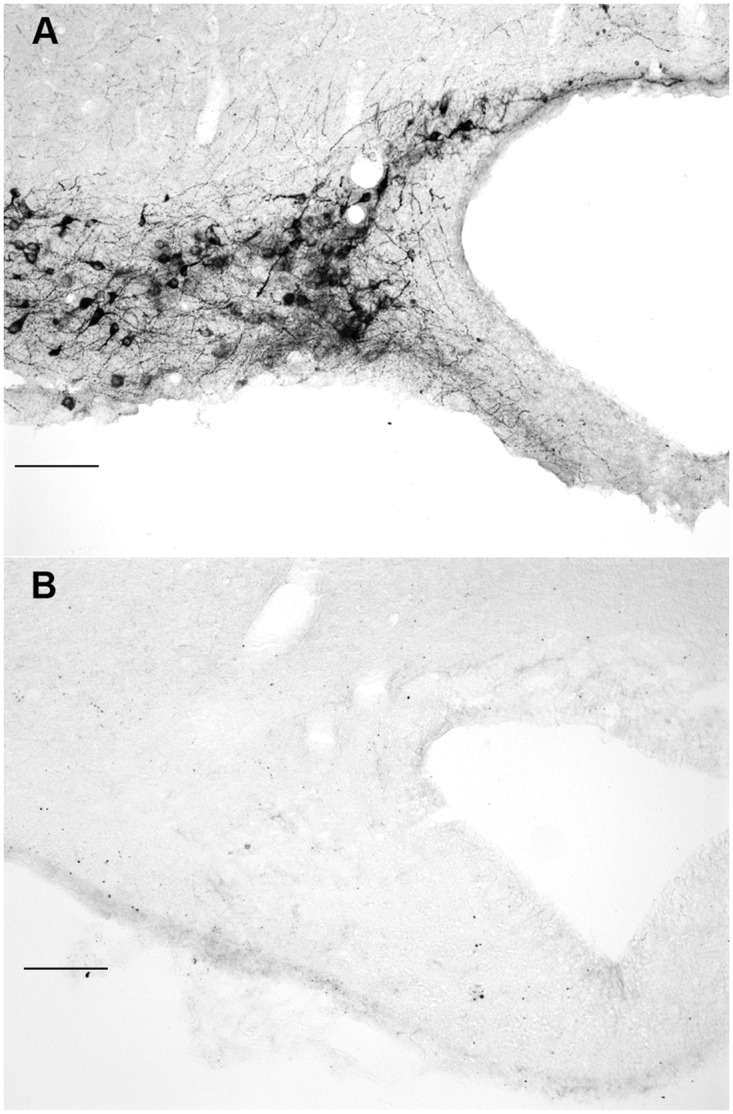
Dependence of histamine immunoreactivity on carbodiimide fixation. Panel A shows histamine immunoreactivity in the vTMN when a brain was perfused and fixed with 4% *N-*(3-dimethylaminopropyl)-*N’*-ethyl carbodiimide hydrochloride followed by 5% paraformaldehyde. Panel B is also of the vTMN in brain that was fixed with only paraformaldehyde. No histamine could be detected. Rabbit anti-histamine antiserum was used at a dilution of 1:20,000. Scale bars = 100 microns.

**Fig 2:**
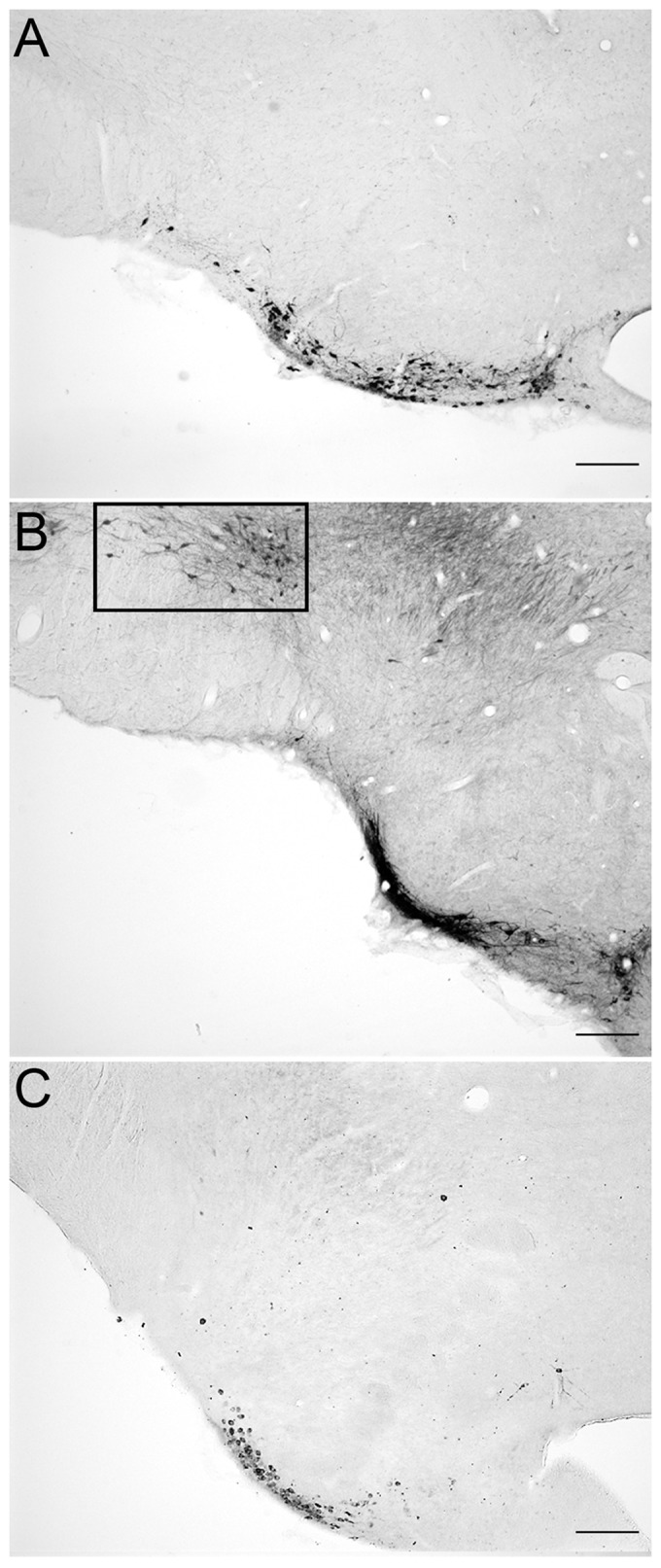
Validation of “true” HDC immunoreactivity in the posterior hypothalamus when optimal antibody dilutions are used and compared to staining of histamine. Panel A shows localization of histamine within the histaminergic neurons of the vTMN from rats perfused with 4% carbodiimide and then 5% paraformaldehyde. There is abundant histamine in soma and in the cell processes. Anti-histamine antiserum was at a dilution of 1:20,000. Panel B is of the same region of the posterior hypothalamus using rabbit anti-HDC antiserum at a low dilution (i.e., high concentration of IgG) of 1:100,000. The *boxed* area highlights significant staining of cross-reacting decarboxylase enzyme(s) in dopaminergic neurons in substantial nigra. In addition, staining occurred within cross-reactive noradrenergic and serotonergic axons in the medial forebrain bundle (*upper right*), and HDC in histaminergic neurons in the ventral aspect of the TMN had intense staining (*lower right*). Panel C shows that when the anti-HDC antiserum was used at a dilution of 1:300,000, staining was selective for the histaminergic neurons of the TMN complex and that cross-reactive staining was eliminated. Scale bars = 100 microns.

Prior to examining HDC immunoreactivity, the location of histaminergic neurons needed to be defined. This approach used tissue fixed with carbodiimide-paraformaldehyde and stained with a validated antibody against histamine. Fig 1, panel A is a high magnification view of the vTMN showing the pattern of histamine immunoreactivity in brain tissue that was fixed with 4% carbodiimide and 5% paraformaldehyde, and using anti-histamine antiserum at a dilution of 1:20,000. Staining for histamine in soma and processes was intense, well-defined, and all immunoreactive cells were confined to the TMN complex. Panel B is of the same location but in brain that was fixed with only 4% paraformaldehyde and stained using identical conditions. No specific histamine was detected when carbodiimide was omitted as part of the fixative, or if acrolein was added to the paraformaldehyde fixative. A lower magnification view of the vTMN of brain fixed with 4% carbodiimide and 5% paraformaldehyde is shown in Fig 2, panel A. Histamine was abundant in soma of histaminergic neurons and was also evident in fibers emanating from them. The dTMN had similar staining but importantly, no other brain regions revealed cell labeling for histamine. Neuronal patterns were consistent with those published previously by others [29, 30].

### Titration of Anti-Histamine Decarboxylase (HDC) Antibody

**Fig 3:**
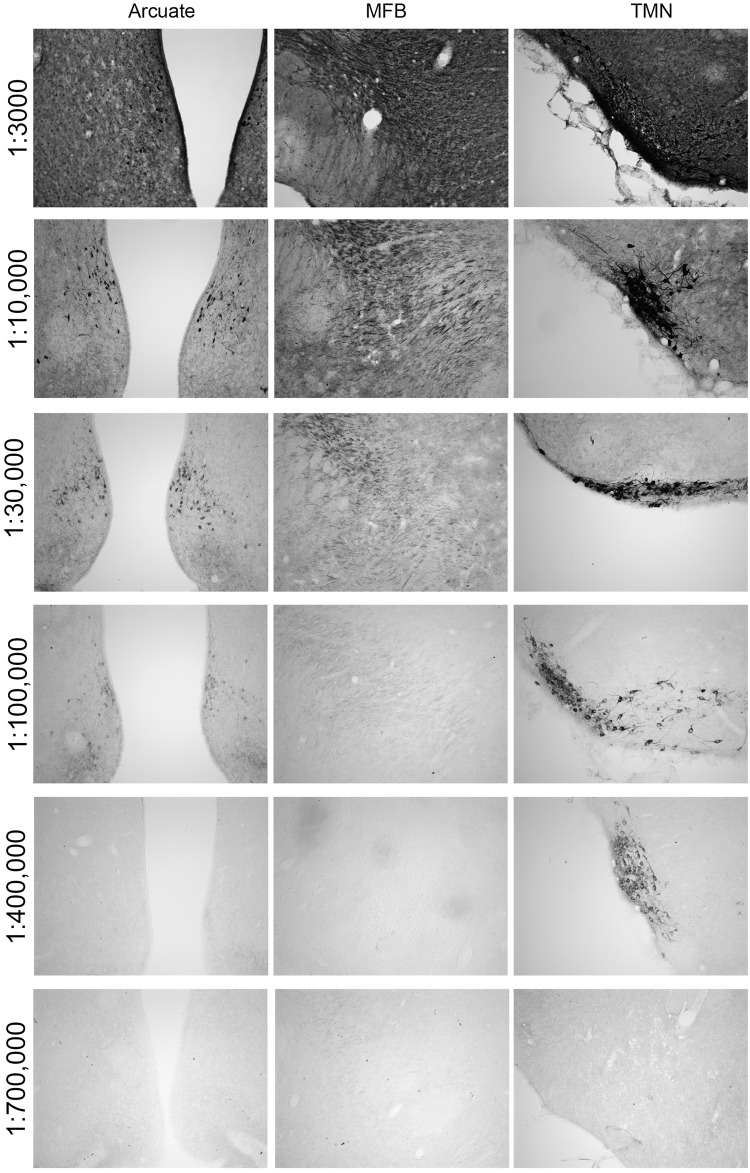
Rabbit anti-HDC antibody titration. Antibody was diluted from 1:3000 (*top* panels) to 1:700,000 (*bottom* panels) using rat sections of the arcuate nucleus that lack histaminergic cells (*left* panel), medial forebrain bundle that lacks histaminergic axons (*middle* panel), and tuberomammillary nucleus where the histamine neurons are located (*right* panel).

**Fig 4:**
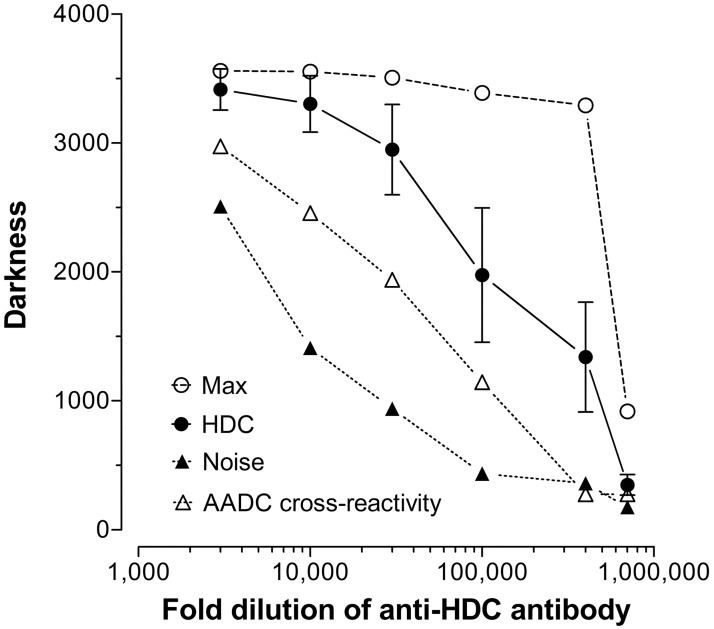
Antibody titration as darkness. Assessment of the staining intensity for histaminergic neurons in the TMN (●), TMN maximal intensity (○), intensity of cross-reactive dopamine neurons (due to L-amino acid decarboxylase [AADC]) in the arcuate nucleus (△), and non-specific background staining, “noise”, (▲) across the spectrum of anti HDC dilutions.

Having established location of histamine-containing neurons of the TM region, the next step was to assess if the staining patterns of the neurotransmitter would be similar for HDC in the same complex. Within these soma, HDC synthesizes histamine from histidine by a decarboxylation reaction. There are reports of cross-reactivity of rabbit anti-HDC antiserum to aromatic L-amino acid decarboxylase (i.e., L-DOPA; EC 4.1.1.26) [44–46], an enzyme of biosynthesis with wide distribution in the brain [50, 51]. It is possible that epitopes in the HDC molecule provoke antibodies that recognize the L-amino acid decarboxylase, but more likely only some of the antibodies generated will provide cross-reactivity. Thus, the advantage of polyclonal antibodies is that the enzyme-specific clones will outnumber the few antibodies that are able to recognize other enzymes. In order to eliminate acquisition of false-positive data, anti-HDC antiserum titrations were performed to optimize staining for only this enzyme and not the cross-reactants. In addition, ICC will not be quantifiable when antibody concentration is too high (saturating product generation), and conversely, may miss targets when antibody concentration is too low [49]. Fig 3 illustrates this principle using anti-HDC antibody at dilutions of 1:3000 (*top*) to 1:700,000 (*bottom*) in two areas that lack histamine: the arcuate nucleus where dopamine neurons are found (*left* panel), and medial forebrain bundle (MFB) where monoaminergic axons from norepinephrine and serotonin but not histamine neurons are located (*middle* panel); the posterior hypothalamus containing the TMN histaminergic system is shown on the *right* panel. At low dilutions to 1:30,000 there is considerable cross-reactivity in irrelevant brain regions such as arcuate nucleus; at high dilutions to 1:300,000–400,000 or more, cross-reactivity is attenuated and eventually lost completely. Importantly, maximal intensity of staining was maintained in the TMN at dilutions where cross-reactivity was minimized or eliminated. Fig 4 illustrates the fold-dilution of anti-HDC antibody when plotted against maximal darkness, specific signal from HDC, background obtained for TMN, and then cross-reactive staining from arcuate neurons (patterns for other non-histaminergic systems were similar to that of the arcuate). As is apparent, reducing antibody concentration drops the noise levels so that from 1:100,000 and beyond, it was minimal. Similarly, cross-reactants in arcuate nucleus were minimal when antibody was used at 1:100,000 or higher dilution. Specific staining for HDC immunoreactivity scored as darkness shows good linearity between dilutions of 1:30,000 to 1:400,000, and then falls considerably at 1:700,000. Maximal intensity is maintained at a nearly constant level between 1:30,000 and 1:400,000. The optimal concentration for this antibody was defined as that amount of antibody that maintained maximal intensity in some structures and provided minimal loss of histaminergic structures but displayed an expected variation in intensity characteristic of biological systems. Moreover, at that concentration, there is no detection of immunoreactive cells or axons in regions of the brain that otherwise show immunoreactivity for non-histaminergic systems such as norepinephrine, serotonin or dopamine that were detected at higher concentration ranges. An example is shown in Fig 2, panel A, which contains a section from a carbodiimide-fixed brain reacted for histamine; panels B and C are similar regions from acrolein-paraformaldehyde fixed brains stained for HDC using antiserum dilutions of 1:100,000 (panel B) and 1:300,000 (panel C). There are four features of interest. First, in panel A of Fig 2, staining for histamine is clear and at this level shows the cells in the ventral TMN and axons emanating from them. No other cells are present at this level and throughout the brain, and are only found in TMN divisions. Second, the *boxed* area in panel B shows significant staining of enzyme(s) in soma of dopaminergic neurons of the substantial nigra at 1:100,000. Axons of noradrenergic and serotonergic neurons in the medial forebrain bundle immediately medial to the *boxed* region are also stained. Third, staining of HDC in histaminergic neurons in the TMN was intense (*lower right*) and was saturated. There also was labeling in neurons of the ventral tegmental area (dopamine), anterior hypothalamus, regions of dopaminergic neurons of the A12-A15 populations, and other cell staining was consistent with descriptions of distributions of aromatic L-amino acid decarboxylase. And fourth, only when the anti-HDC antiserum was diluted to 1:300,000 to 1:500,000 (shown in panel C at 1:300,000) did the staining reflect selectivity for the histaminergic system with cell localization confined to neurons in the TM complex and axon labeling associated with those cells. All cross-reactive staining in regions outside of the TMN was eliminated, even when the staining in the histaminergic system was strong as shown. Thus, panels A and C of Fig 2 illustrate specificity where the patterns for histamine and HDC immunoreactivity, respectively, are practically identical and are localized to the TM complex and its projections.

### Alterations in HDC by Chronic REM-SD

**Fig 5:**
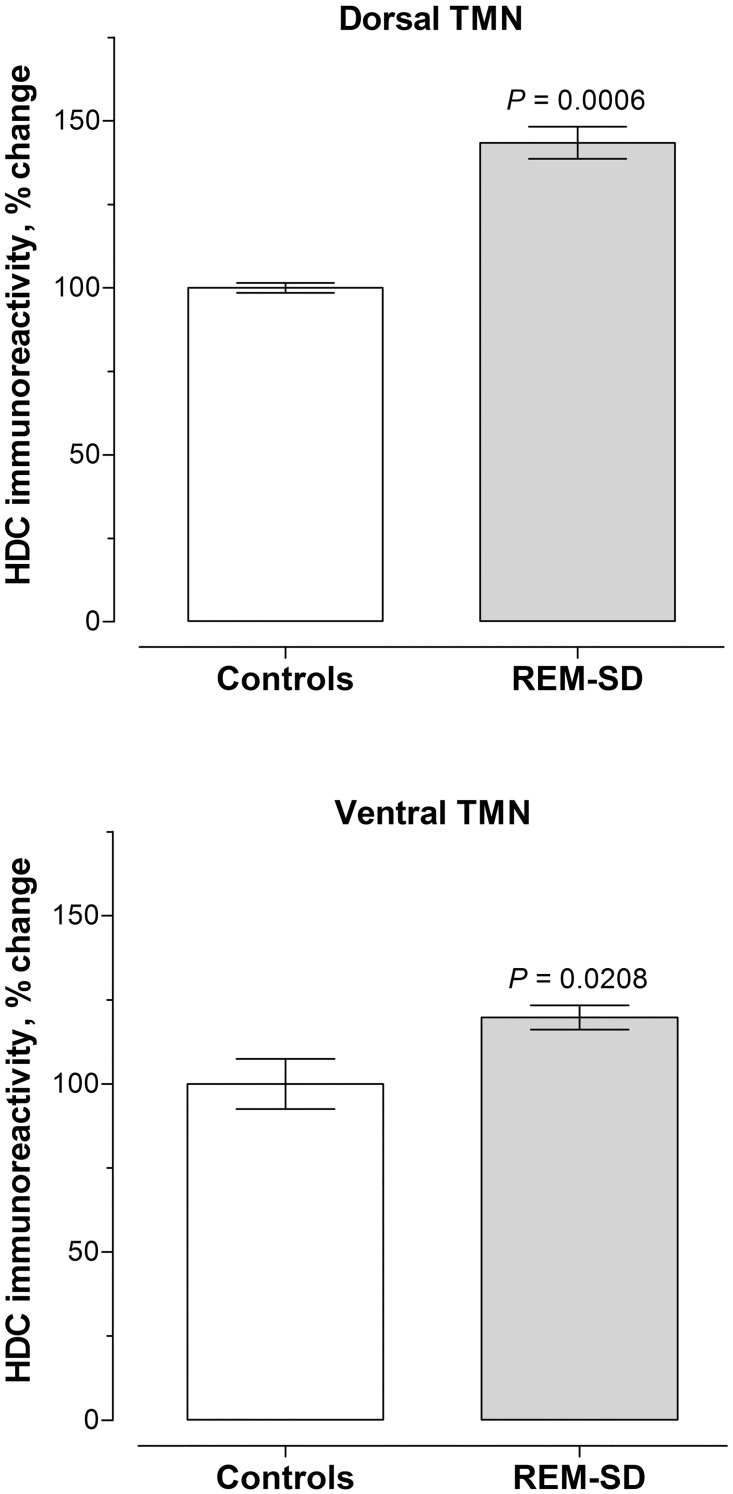
Changes in HDC in the TM complex with REM-SD. Immunoreactivity of HDC in control and sleep-deprived rats was scored as darkness of staining minus background in dTMN and vTMN, and is presented as a percent change from controls. Data of dTMN and vTMN from 5- and 10-day sleep-deprived rats were pooled for each population of histaminergic neurons because beyond *day* 5, HDC immunoreactivity remained high and was not significantly different. Compared to controls, REM-SD significantly increased HDC in dTMN by 58 ± 6.5% (t = 4.631; *P* = 0.0003); *upper* panel) and in vTMN by 19.8 ± 3.6% (t = 2.741; *P* = 0.0208; *lower* panel). See also S1 Data.

Satisfied that the anti-HDC antiserum at 1:300,000 was appropriate to specifically label the histaminergic system and not cross-reactants, brain tissue of rats from a previous experiment of chronic REM-SD was examined [20]. Intensity of immunoreactive HDC in both areas of the TMN increased significantly by *day* 5 and remained elevated. Because HDC did not change for the duration of the REM-SD regimen (i.e., for *days* 5 and 10; dTMN, t = 0.840, *P* = .4425 [the 2 rats from the *day* 15 cohort were not analyzed due to its small sample size]; vTMN, t = 0.252, *P* = 0.809), the REM-SD data as *days* were pooled. Fig 5 graphs the percent changes in darkness of HDC immunoreactivity in neurons of the dTMN (*upper* panel) and vTMN (*lower* panel) of REM-SD rats compared to home cage controls (see S1 Data. Raw data underlying Fig 5). The experimental regimen resulted in significantly increased HDC immunoreactivity in both neuronal populations of the TMN: dTMN increased by 58 ± 6.5% (t = 4.631; *P* = 0.0003) and for vTMN, the increase was 19.8 ± 3.6% (t = 2.741; *P* = 0.0208).

**Fig 6:**
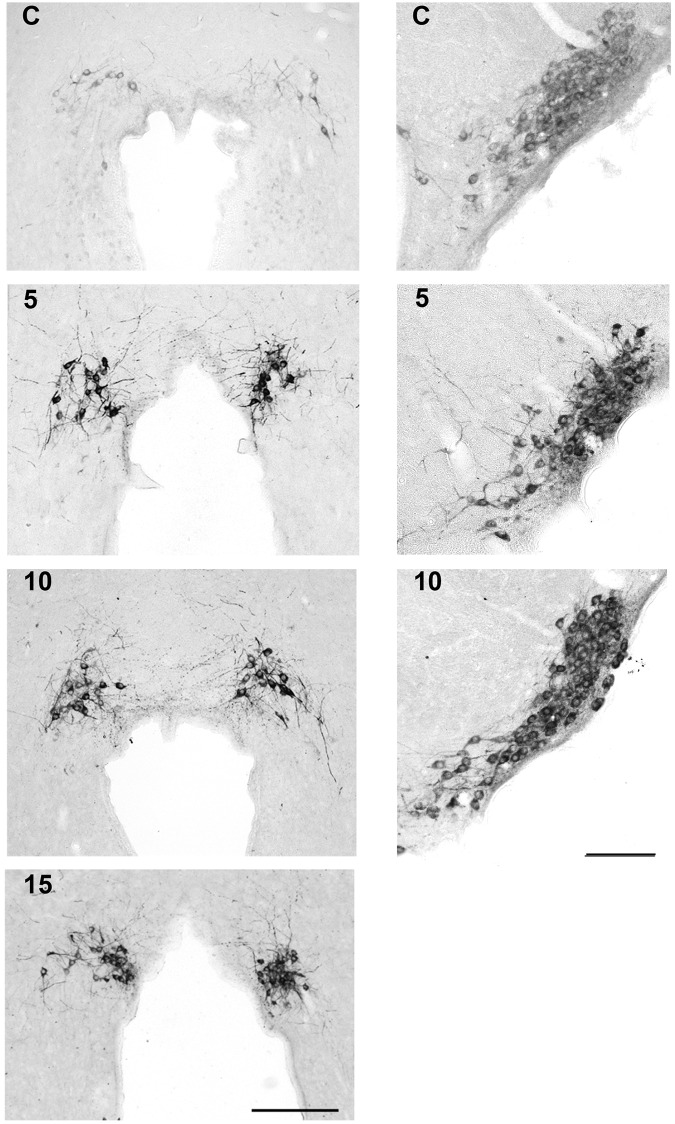
Changes in HDC immunoreactivity in the TMN complex of representative control and REM-SD rats. *Left* panels show HDC in dTMN of a control rat and those undergoing REM-SD for 5, 10, and 15 days. The *right* panels show HDC in vTMN for control and REM-SD for 5 and 10 days. Scale bars = 100 microns.

**Fig 7:**
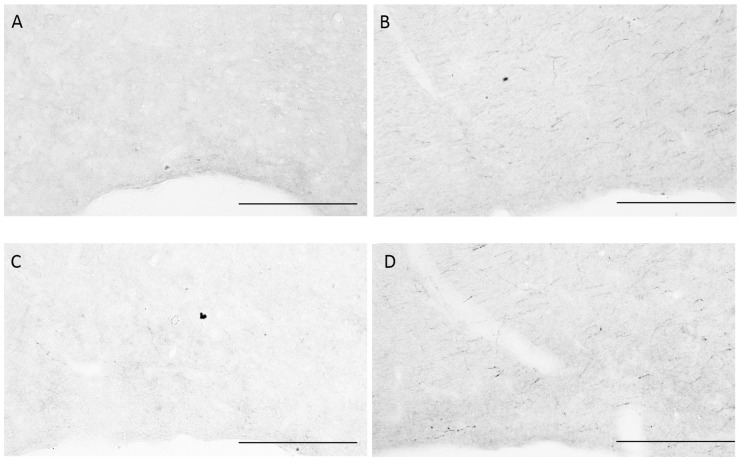
HDC in histaminergic fibers within the VLPO. Axons from the histaminergic system are shown in VLPO sections from two control (panels A and C) and two 10-day REM-SD rats (panels B and D). The intensity of HDC in axons increased in the REM-SD rats. Scale bars = 25 microns.

Histological changes in HDC immunoreactivity are shown in Fig 6 from representative sections. The panels on the *left* are of HDC in dTMN at low magnification. In a home cage control rat (*top* panel labeled C), there was a modest amount of HDC in neurons around the superior aspect of the posterior 3^rd^ ventricle. By *day* 5 of REM-SD, a substantial increase in HDC had occurred in soma and their processes. Robust up-regulation of HDC was maintained for the duration of the experiment to *days* 10 and 15. The panels on the *right* are of HDC in vTMN at a higher magnification. In examining home cage controls, it is apparent that there was more immunoreactive HDC in vTMN compared to dTMN. Nevertheless, HDC significantly increased after 5 and 10 days of REM-SD, although the magnitude of change was less compared to the dTMN (see Fig 5). HDC immunoreactivity was also examined in sections of the ventrolateral preoptic nucleus (VLPO;Fig 7), a target of histaminergic projections. In representative home cage control rats, there are no discernible HDC-positive fibers (panel A) or just a few (panel C). By 10 days of REM-SD, however, the number of fibers immunoreactive for HDC increased (panels B and D). The purpose of Fig 7 is only for visual inspection; statistical analysis was not done due to small sample size.

## Discussion

This study has two significant outcomes. First, a methodological component complements and reinforces earlier studies. In 1984, Panula described that antibodies raised using an immunogen of histamine conjugated to hemocyanin with carbodiimide identify histaminergic neurons as being localized in the posterior hypothalamus [52]. In addition to its properties as a conjugant, 2% carbodiimide proved to be a good fixative for immunohistochemistry [53], but staining improved if tissues were fixed with carbodiimide at 4% followed by 4% or 5% paraformaldehyde as a post-fixative [54]. Irrespective of some differences in methodological procedures, neuroanatomical studies of mammalian taxa involving mouse, rat, guinea pig, and tree shrew show prominent histamine immunoreactivity for cell bodies in the posterior hypothalamus, now recognized as being the tuberomammillary nuclear (TM) complex [29, 55, 56]. It is also apparent that composition of the brain fixative solution is critical. Specifically, if 4% paraformaldehyde is used alone or with addition of 2.5% acrolein, immunoreactivity of histamine is poor, and this study emphasizes initial fixation with carbodiimide followed by paraformaldehyde to achieve satisfactory staining.

Earlier immunocytochemical studies to localize L-histidine decarboxylase (HDC), the enzyme responsible for histamine synthesis, revealed significant cross-reactivity to aromatic L-amino acid decarboxylase [44–46], an enzyme of monoamine biosynthesis found throughout the brain [50, 51]. The cross-reactivity is most likely due to common antigenic determinants of these enzymes based on cDNA sequence analysis [46]. In any case, within the TM complex, there is immunoreactivity with anti-HDC antisera [44–46] in the same regions that *in situ* hybridization reveals HDC mRNA [42, 43]. In guinea pig, HDC immunoreactivity after formalin fixation is found in both populations of TMN neurons and cross-reactive decarboxylase staining in areas beyond those validated for histamine neurons [46]. This attribute appears true for our rat study and may reflect the enhancement of immunoreactivity for most peptides and proteins by 2.5% acrolein added to buffered 4% paraformaldehyde [38, 48], together with the overall increases in sensitivity of the immunocytochemical procedures employed by our laboratory. Importantly, by titration of the primary antibody, objective measures of staining intensity defined the linear portion of the detection curve, and defined the optimal range for HDC detection to permit measurable increases (or decreases) in protein expression. Dilution of anti-HDC antiserum used in our study to at least 1:300,000 was necessary to eliminate the previously reported cross-reactivity and yet have appropriate specificity and sensitivity to observe HDC in the TM complex.

The second outcome of this study is that chronically REM sleep-deprived rats appear to increase the capacity to synthesize histamine by increasing HDC within the TM complex. Consequently, there was more immunoreactive HDC detected within fibers of the VLPO, to where histaminergic neurons of the TM complex project. In some immunocytochemical assays of axons, numbers of objects may not differ, but the size of detected axons changes [57]. We did not measure release of histamine or monitor activity of histaminergic neurons of the TMN in any of our rats because the earlier experiment [20] was designed for brain perfusion. At the same time, the evidence that histaminergic neurons in the TMN have a major role in arousal and wakefulness is unequivocal [33, 58–60]. Many studies have corroborated histamine’s involvement in experimental sleep deprivation or restriction. For example, levels of *tele*-methylhistamine, a metabolite of neuronal histamine, become elevated in rats when subjected to the flowerpot paradigm for 72 hours [61]. This suggests that forced wakefulness increases histamine release. Histamine in cerebrospinal fluid is elevated when rats are awake, briefly sleep-deprived for 6 hours, or when treated with a histamine-3 receptor antagonist that promotes wakefulness [32]. During 6 hours of sleep deprivation of rats by placement of novel objects in cages, *in vivo* microdialysates of basal forebrain revealed rapid and sustained increases in histamine for the duration of the experiment, and tight correlation between histamine and percentage of time in wakefulness [62]. Gentle handling of cats for 6 hours to enforce sleep deprivation causes a rise in histamine in microdialysate samples from the preoptic anterior hypothalamus [63], the presumptive sleep center [64]. The high histamine levels are comparable to those found during normal wakefulness, which can be explained as an inhibitory effect on sleep-promoting neurons of the preoptic anterior hypothalamus. These studies point to release of histamine being pivotal in keeping subjects awake when sleep is denied. The findings are consistent with the understanding that in monoaminergic systems, increased neurotransmitter release and up-regulation of the enzymes responsible for their synthesis go hand-in-hand (e.g., [27, 28]). More specifically for the histaminergic system within the hypothalamus of acutely stressed rats, there is increased histamine [65–67], increased histamine turnover [68], and elevated HDC activity [67]. We posit that the increased expression of HDC during chronic REM-SD suggests greater synthesis of histamine, which may explain how rats can stay awake in the deprivation enforcement chambers despite a need to sleep.

It should be mentioned that histaminergic neurons of the TMN are organized into 5 E-groups [69], and on the basis of c-Fos activation and HDC mRNA detection following various stressors, the more stress-sensitive histaminergic neurons comprise the E4-5 regions in the dorsal aspect of the TMN [70]. We found higher HDC immunoreactivity in the dorsal TMN compared to the ventral aspects during REM-SD (see Fig 6), and in our previous study involving the same cohort of rats [20], corticotropin releasing hormone (CRH) gene expression in the paraventricular nucleus significantly increased by *day* 5 and remained elevated. These data involving CRH and HDC (in E4-5 regions of dTMN) suggest activation of the hypothalamic-pituitary-adrenocortical stress axis.

An outcome observed with chronic REM-SD studies of rats using the flowerpot paradigm and its many variants is hyperphagia [22] and elevated energy metabolism and expenditure [17–19, 71, 72]. The present data show that levels of HDC, especially in the dTMN, reflect increased capacity to synthesize histamine and are likely linked to pathways evoking hyperphagia. One action of histamine is to stimulate activity of the sympathetic nervous system [73], which in turn increases sympathetic outflow to BAT [36, 37] and triggers thermogenesis [35]. The increase in metabolic rate will initiate negative energy balance that should promote appetite. This introduces a remarkable but challenging *yin-yang* dichotomy because evidence points to histamine being an anorexigenic signaling neurotransmitter [74]. Histaminergic control is complex and includes leptin [75], CRH [76], nesfatin-1 [77], and thyrotropin releasing hormone (TRH) [78], all of which are peripheral or central satiety signals. The blunting of appetite via histamine appears to be initiated by leptin [79]; however, since circulating levels of leptin decrease significantly in sleep-deprived or restricted rats [14, 17, 19, 80], the hyperphagic response is not surprising. At the same time, our finding of up-regulated HDC appears to pose a conflict that has similarities to the persistently elevated hypothalamic CRH observed in chronically REM sleep-deprived rats [20]. If both histamine and CRH promote anorexia, how is it that sleep deprivation consistently results in hyperphagia? As we previously speculated [20], it is possible that the anorexic effects of these neurotransmitters may be inhibited or overridden, or that there may be a change in processing of satiety signals because of the state of elevated energy metabolism and expenditure that ensues with chronic sleep deprivation and/or restriction [17–19, 71, 72] and the need for hyperphagia to fuel this response. This aspect may have credence based on our observations that following euthanasia, sleep-deprived rats usually have distended stomachs and had often been eating prior to termination of the experiment.

Current understanding of histamine projections and their functions favors the notion that they serve as a focal point for the regulation of not only being awake, but also behavioral and metabolic changes. For instance, high rates of histamine synthesis and release could contribute to the hyperactivity that prevents immediate sleep when a subject no longer is barred from sleeping. In rats, this phenomenon is likened to mania [81]. Since histaminergic systems project to a variety of other brain regions such as cerebral cortex, forebrain areas involved in autonomic control, and brainstem areas beyond the reticular activating paths [82, 83], additional functions could be influenced by alterations in histaminergic tone. An intriguing example is that histamine axons extend to the mesencephalic trigeminal nucleus where mastication reflexes are coordinated [84], and an elevated histamine release at this site could account for sleep-deprived rats manifesting aberrant gnawing behavior of inedible materials [85].

An interesting model to consider in piecing together these various bits of information is that increased histamine release may stimulate peripheral sympathetic activity [73] via histaminergic projections to the hypothalamic dorsomedial nucleus and the intermediolateral cell column of the spinal cord [86]. As Yasuda et al. demonstrated, increased histamine may be the trigger to elevate energy metabolism by activating UCP1 in BAT [36, 37], which begins to clarify the mechanistic pathways governing sleep deprivation hypermetabolism [17–19, 71, 72]. Thus, we suggest that the histaminergic systems operate to reinforce the continuous need to maintain wakefulness during chronically enforced REM-SD as well as to alter sympathetic tone that in rodents increases metabolism, leading to problems in weight homeostasis and other pathologies.

## Supporting Information

S1 Data Raw data to accompany Fig 5. Measured darkness was converted to percent changes from controls.
(PDF)
