# Supplementary material for: Hypothalamic L-Histidine Decarboxylase Is Up-Regulated During Chronic REM Sleep Deprivation of Rats
Source: PLoS One. 2016 Dec 20;11(12):e0152252. doi: 10.1371/journal.pone.0152252 (PMC5172538; doi:10.1371/journal.pone.0152252)
Supplement: S1 Data — Measured darkness was converted to percent changes from controls. (PDF) [file pone.0152252.s001.pdf]

Authors: [Gloria E. Hoffman]
[Michael Koban*]   * corresponding author

Title: **Hypothalamic L-Histidine Decarboxylase is Up-Regulated During Chronic REM Sleep Deprivation of Rats**

Raw data to accompany Figure 5. Measured darkness was converted to percent change from controls

	Control, dTBM	REM-SD, dTBM	Control, vTBM	REM-SD, vTBM
	78.7	167.4	80.2	113.0
	104.6	121.3	105.0	126.1
	110.3	159.1	115.9	134.3
	106.4	139.6	98.9	109.5
		176.5		121.4
		133.7		104.0
		145.3		122.8
		155.4		126.9
		160.4		
		159.6		
		170.9		
		148.6		
		216.7		
**Means:**	100.0	158.0	100	119.8
